# Social network characteristics and alcohol use by ethnic origin: An ego-based network study on peer similarity, social relationships, and co-existing drinking habits among young Swedes

**DOI:** 10.1371/journal.pone.0249120

**Published:** 2021-04-08

**Authors:** Nina-Katri Gustafsson, Jens Rydgren, Mikael Rostila, Alexander Miething

**Affiliations:** 1 CHESS, Department of Public Health Sciences, Stockholm University, Stockholm, Sweden; 2 Department of Sociology, Stockholm University, Stockholm, Sweden; Emory University, School of Public Health, UNITED STATES

## Abstract

The study explores how social network determinants relate to the prevalence and frequency of alcohol use among peer dyads. It is studied how similar alcohol habits co-exist among persons (egos) and their peers (alters) when socio-demographic similarity (e.g., in ethnic origin), network composition and other socio-cultural aspects were considered. Data was ego-based responses derived from a Swedish national survey with a cohort of 23-year olds. The analytical sample included 7987 ego-alter pairs, which corresponds to 2071 individuals (egos). A so-called dyadic design was applied i.e., all components of the analysis refer to ego-alter pairs (dyads). Multilevel multinomial-models were used to analyse similarity in alcohol habits in relation to ego-alter similarity in ethnic background, religious beliefs, age, sex, risk-taking, educational level, closure in network, duration, and type of relationship, as well as interactions between ethnicity and central network characteristics. Ego-alter similarity in terms of ethnic origin, age and sex was associated with ego-alter similarity in alcohol use. That both ego and alters were non-religious and were members of closed networks also had an impact on similarity in alcohol habits. It was concluded that network similarity might be an explanation for the co-existence of alcohol use among members of peer networks.

## Introduction

People’s social contacts and networks have been found to have an impact on their norms and behaviours. It is for example well known from individual-level studies that alcohol habits are socially patterned, and that family, friends, and other people in the extended network influence people’s drinking behaviours. Although people hold individual norms about behaviours, these are often revised during such interactions. This influence is, furthermore, bimodal since networks can have both positive and negative impact on people’s drinking behaviour [1] i.e., social interactions can trigger people to drink both more and less. Peer pressure have been observed to be a strong predictor of young people’s alcohol use, but the association is mediated by intrapersonal predictors such as self-esteem, health locus of control and receptivity to peer pressure [2]. It is also dependent on the situational aspects (situational norms and contextual setting). In settings where drinking is encouraged by peers and/or the environmental setting, people are more likely to drink [3,4].

Little is, however, known about what aspects of these relationships that is associated people’s drinking. It has been suggested [5] that a person’s health behaviours can have collateral health effects i.e., externalities, also among his/her peers and increase the risk/chance of a similar behaviour in the other. It has been suggested that the impact is stronger among peers who resemble each other [6]. This could furthermore result in a change (or spread) of behaviour from one person to another i.e., induction, which have been observed to be stronger in closer ties [6].

Dyads are the smallest entity in a network, and these are the most immediate level of peer interaction. It has for example been observed in psychological experimental settings that people in dyads tend to ‘imitate’ the drinking level (non/light/heavy) of the other peer [7]. Most dyads are, however, nested within larger peer networks and also more distant ties could alter the behaviours [8]. Peer pressure is also a common explanation, particularly in relation to young people’s drinking [9]. A dyad between two close peers is, however, expected to involve more direct or deeper communication, which is not always the case between peers that are part of a larger network. For this reason, the most important dyads are expected to have a greater influence on norms and behaviours than other peers have. In fact, it has been observed that measures of dyad similarity rarely are strongly related to measures of heterogeneity in complete networks [10]. Since it is assumed that peers’ part of a dyad influence each other both ways, their behaviours could further be seen as an average of both persons’ initial behaviour.

Homophily e.g., similarity between peers, is known to be strong in dyads. This has for example been observed regarding demographic characteristics such as ethnicity, social class, and gender [10,11]. Similarity in ethnic background has in fact been argued to be the strongest divides of social life and particularly important for people’s attitudes [10]. In this regard, similarity could potentially increase the likelihood of peers being more prone to also influence each other’s health behaviours e.g., alcohol habits. Although it has been much debated whether people select friends based on homophily or adapt to their peers’ drinking habits (assimilate) [12,13]; it seems to be a combination of these two. Peers already prone to drinking tend to self-select into peer groups with other drinkers [14] and it is likely that alcohol use occurs both as a result of similarity and of assimilation [15]. It has also been argued [16] that although everyone at some point is selected, a major part of people’s substance use is influenced by other people’s use. While alcohol habits change and co-evolve in peer networks, it is likely that the strong similarity between peers make them more prone to change together. Dyadic (pairwise) processes between two peers tend to be two-sided, thus behaviours vary depending on with whom a person interacts and the behaviour of that person [17]. There is currently no well-defined theory about homophily and its contribution to inequalities in health (risk) behaviours or health outcomes, but a few studies have shown that similarity among peers could predict e.g., diet, physical activity and smoking behaviour [18–20]. At least smoking has been found to spread more easily if egos and alters are similar to each other [21]. Other network characteristics, related to how close peers are to each other and how often they meet, are also likely to have importance for drinking habits in terms of how likely they are to influence each other’s behaviours [8,18,22,23].

Another interest of this study is to investigate the role of ethnic background for drinking habits in social networks. The number of people with migrant background is fairly large in Sweden and has furthermore increased during later years (Statistics Sweden). Alcohol habits are known to vary largely by national drinking culture, but also to differ between first- and second-generation immigrants in the destination country [24,25]. Little is known on how migrant background is associated with alcohol habits in peer relationships.

### Norm based mechanisms

When people are very similar to each other regarding personal characteristics e.g., if they have similar ethnic background, share similar religious beliefs, have the same gender and age, it is likely that they also share similar attitudes and norms about certain behaviours e.g., alcohol habits.

Alcohol habits are known to differ between various drinking cultures, expressed as differences in what situations alcohol is consumed (e.g., with/without meals), total volume of alcohol consumed, or volumes consumed on one occasion (binge drinking), but differences in alcohol cultures are at least in part also related to religious beliefs [26]. The traditional Swedish drinking culture has supported a drinking pattern more centralized to weekends and holidays, often including binge drinking [27]. The decrease in alcohol consumption which have been observed in Sweden compared to the peak in 2004 [28] has been suggested to be related to a change in norms related to the increasing number of first- and second-generation immigrants from countries with disparate drinking cultures to the traditional Swedish one. In this study, young adults with Swedish-born parents are compared to those with either parent born in former Yugoslavia or Iran. The two latter countries are representing drinking cultures disparate from the Swedish one: the habits of former Yugoslavia representing a more continental drinking of wine/beer with meals and higher consumption levels but less binge drinking than the Swedish tradition, and Iran representing a more restrictive drinking culture partly restrained by religious beliefs.

Parents are expected to keep some of their traditional norms e.g., alcohol culture, also in the new country [29,30]. Since parents drinking tend to have an impact on young peoples’ drinking through intergenerational socialization processes [31], it is furthermore assumed that the drinking habits of the young people to some extents are influenced by the drinking culture in their parents’ country of origin. It is expected that alcohol use is less common among children of parents from countries with stronger cultural and religious norms that forbid alcohol use as a result of the socialization at home. At the same time, second generation migrants might be socialized into the drinking culture in the “new country” and acculturate towards these habits. For diet it has e.g., been observed that second generation migrants eat less healthy than their parents and that obesity is more common i.e., the Hispanic paradox [32]. A further implication of different drinking cultures and habits in the parent’s countries of origin is that there might be preferences for certain beverages e.g., vodka/strong spirits over table wine. It is also likely that the young people cluster with peers sharing similar background due to selection processes, further increasing the likelihood of them sharing the same norms and habits. Ethnic background might additionally co-vary with other dimensions e.g., socio-economic status [10]. The tendency for people to spend time in homogenous networks (with peers similar to themselves) is therefore expected to work in favour of maintaining traditional habits.

Still, also within one drinking culture, there can be variations in norms e.g., by population groups [33]. It is well known from individual-level studies that young people drink more than older people [34,35] and that men drink more than women [36]. It seems like different norms applies to different population groups which result in different habits. It is e.g., less accepted that women drink large amounts of alcohol [37], or that older people binge drink as often as young adults [38]. Norms of drinking are not as explicit when it comes to socio-economic status, although historically drinking in the working class have been more controlled since this group was argued to have a more harmful drinking pattern and to prefer beverages with higher alcohol content [39]. There is also a strong gender homophily among peers, with a preference for same sex relationships in closed networks [15]. Here it is expected that two people sharing several characteristics are also more likely to both be drinkers given that demographic homophily is strongly associated with the spread of information and behaviours.

### Contact opportunities enhancing norms and norm-based behaviours

Although wider networks have been suggested to influence a person’s alcohol habits, as is the case when alcohol habits are spread in societies and form collective (national) drinking cultures [40], the immediate network seems to have greater impact on people’s habits [1]. The main argument for this is that close peers have more contact opportunities. People drink like their peers [41,42] and other people they often spend time with e.g., parents [43,44] or romantic partners [45]. It has even been suggested that epidemics in health risk behaviours e.g., alcoholism, might develop as a result of peer-to-peer impact [5,6]. An increasing peer interaction has also been observed to be important for explaining other health risk behaviours e.g., onset and continuation of smoking [46,47]. The strength of a peer relationship depends on how much time they spend together as well as on the quality of the relationship e.g., indicated by discussions of private nature. People who often spend time with each other and have a close relationship are more likely to influence each other’s norms and as a result the behaviours, thus closer friends are expected to have a greater impact on a persons’ drinking than an acquaintance. Type of relationship [10] could, furthermore, have an impact on patterns of drinking i.e., if peers binge drink together or not.

Theories of homophily imply that social interactions occur more often between similar people than among dissimilar people [10]. People have greater contact opportunities generally if they share the same friends (i.e., network closure) or specifically if they both spend time in contexts where alcohol consumption is common (e.g., attending same university or activities). Network closure has been suggested to be one aspect which strengthens and maintains health norms and health-related behaviours in networks; depending on the norms it can both have a negative or positive impact on the health behaviours [48,49]. It is expected that peer groups with restrictive norms, which are homogenous and where everyone knows each other, are more likely to create and uphold restrictive views on alcohol. Norms encouraging alcohol use are contradictory re-enforced within networks holding a positive attitude to alcohol. At the same time, dynamic processes have been suggested to be important when people change their behaviours, and then people are more likely to adapt to people who are like themselves.

The intention with this study is to fill a research gap by considering how peer similarity and other network characteristics relate to health behaviours e.g., alcohol use, in relation to disparate ethnic origin. Thus, in this study the homophily concept has a broader definition than what is the common use by not only focusing on socio-demographic similarities but by also considering similarities in behaviours.

### Aim

The objective is to study whether homophily in dyadic relationships—by ethnic background, religious beliefs, gender, age, and social behaviours—and other social network aspects co-exist with peer similarity in alcohol habits.

It is hypothesized that 1) peer similarity in alcohol habits is related to other peer similarity i.e., behaviours are norm-based (culture); that 2) alcohol use is more common and consumed in higher quantities (binge drinking) in homogeneous peer dyads representing population groups commonly known to have higher use e.g., men, younger people, risk-takers; and that 3) alcohol use is more similar among friends with greater contact opportunities with their peers e.g., in close or closed peer ties

## Materials and methods

### Data collection

The data used in this study was derived from a survey entitled “*Social Capital and Labor Market Integration*: A Cohort Study”, a randomly selected representative sample of Swedish residents born in 1990. The strategic sample of respondents (n = 5,695) comprised Swedish citizens stratified by the most common ethnic origins in this cohort. Respondents with at least one parent from former Yugoslavia refer to 50% of this subgroup in the total population in Sweden. Those with at least one parent from Iran comprise the entire ethnic subgroup. A simple random sample of descendants of Swedish parents represents 2.5% of the total population group at that age in Sweden. This sample was representative of the groups included in the analyses, of which 49.4% had two Swedish-born parents, 21.5% had Iranian descent (13.4% both parents, 4.5% had one Iranian parent and one Swedish parent, 3.6% had one Iranian parent and one parent with other descent), and 29.1% were descendants from former Yugoslavia (21.3% both parents, 5.1% had one Yugoslavian parent and one Swedish parent, 2.7% had one parent from former Yugoslavia and one parent with other descent).

**Fig 1:**
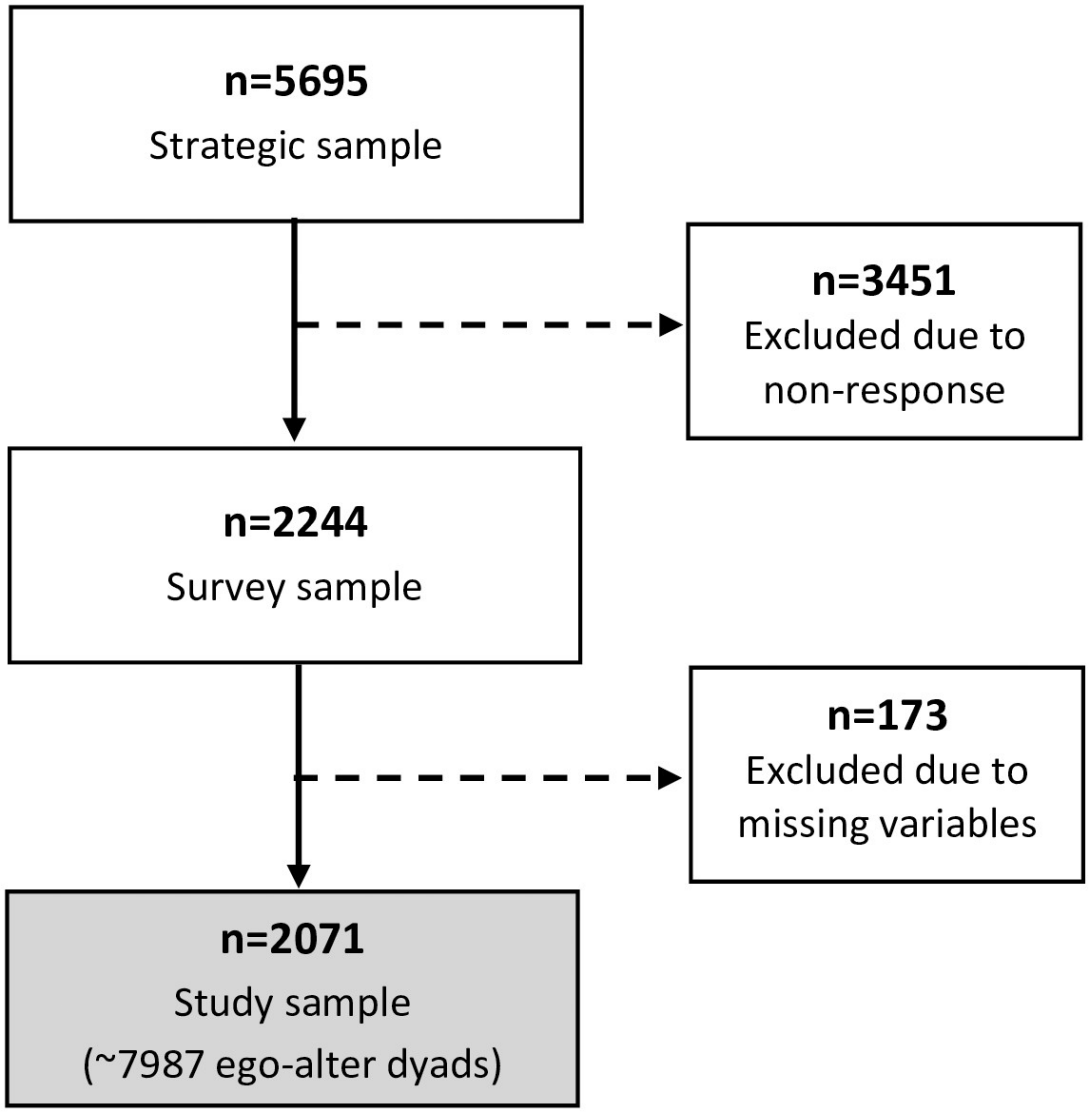
Flow diagram of study sample selection.

From the strategic sample of n = 5,695 in the first wave, n = 2,244 was re-interviewed by telephone by Statistics Sweden in 2013. The response rate was 39.4 per cent in this wave. The widespread use of non-registered prepaid phones in this specific age group was the primary reason for non-responses in the study. The three groups with distinct ethnic background also represent different drinking cultures i.e., habits with restrictive alcohol use and habits including regular binge drinking. Only data from the second wave of the survey (2013) was considered for the purpose of this study since it contained all variables of interest i.e., information on alcohol habits and ethnic background for individuals and their peers, and information about peer network characteristics; respondents were then around the age of 23. After excluding the non-responses on the variables of interest, 2,071 individuals remained in the analytical sample of the present study (Fig 1). These all gave informed active consent to participate in the study. Those living in urban areas, having lower school grades, no-upper secondary or university education, and those whose parents had a lower educational degree had a slightly lower response rate.

For the purpose of analysing dyads, respondents (egos) were asked to name up to five persons (alters) who they considered to be their closest peers. These nominations covered different types of relationships, and a distinction was made between friends, family members and romantic partners. Egos nominated four peers (alters) on average, which resulted in a total of 7,987 ego-alter pairs (dyads) to be included in this study. Since a friendship is intrinsically dyadic and depends on both peers to persist, these dyads are the basis for the analyses. By using a dyadic design, both features of the ego and alter are used to estimate their association toa behaviour. Estimations of social behaviours, such as use of alcohol, are more reliable if they are based on the social ties through which most alcohol use occurs i.e., not only ego’s characteristics but of their peer’s as well. Data were in long format, including multiple observations (up to five observations) per ego (egos can thus be part of several dyads). Composite variables at the dyad level to account for the similarity and dissimilarity in ego-alter ties were constructed; if both ego and alter answered that they had an Iranian parent they were coded as Iranian but if only one of them had an Iranian parent, they were coded as of mixed origin. Thus, all variables refer to similarities in behaviours and attributes in these dyads (pairs) or of characteristics describing the relationship between egos and alters (Table 1).

**Table 1:** Distribution of social network characteristics at the dyad level.

Ego-Alter ties	Frequency (n)	Percent (%)
(0 Baselevel): E&A with **incongruent drinking habits** (one of them do not drink)	1,296	16.2
(1) E&A **do not drink alcohol** (congruent abstainers)	508	6.4
(2) E&A **drink alcohol** (congruent prevalence of alcohol use)	5,451	68.3
(3) E&A **weekly binge drinkers** (congruence, intoxicated at least once a week during last 12 months)	732	9.2
Sex composition		
Female-male	1,479	18.5
Female-female	3,113	39.0
Male-male	3,395	42.5
Similar age (+/- 3 years)	6,858	85.9
Ethnic background (parental country of origin)		
Dissimilar ethnic background	1,872	23.4
Swedish	3,852	48.2
Iranian	1,177	14.7
Yugoslav (former)	1,086	13.6
Similarity in religious beliefs		
E&A disparate confessions	2,778	34.8
E&A neither religious	3,930	49.2
E&A both Christian (Protestant/Catholic)	709	8.9
E&A both Muslim	477	6.0
E&A both other type of belief	93	1.2
E&A both risk-takers	1,892	23.7
E&A both started tertiary education	3,302	41.3
Type of peer-relationship		
Relatives	820	10.3
Romantic partners	513	6.4
Friends (not relatives or romantic partners)	6,654	83.3
Relationship duration in years (mean (standard deviation))	8.4 (6.6)	
E&A in closed networks (closure)	5,929	74.2
Conversation subjects		
E&A discuss important matters with each other	6,999	87,6
E&A talk about religion	4,200	52.6
E&A talk about sex	5,936	74.3
Observations (ego-alter ties)	7,987	100
Individuals (egos)	2,071	

#### Dependent variable

The dependent variable was constructed from two items on ego’s drinking habits 1) prevalence of alcohol use i.e., egos answered an item on whether they drink alcohol or not, and 2) weekly binge drinking, constructed from an item asking how many times ego had been drinking enough alcohol to feel intoxicated during the last 12 months (positive responses to the alternatives ‘Three times a week or more often’ and ‘Once to twice each week’) was recoded to ‘binge drinking at least weekly’, and corresponding items for alters drinking. The final variable used in these analyses was a composite measure of similarity in prevalence of alcohol use and frequency of weekly binge drinking in the ego-alter dyads (peer ties). It had four categories: 1) disparate drinking habits in dyads (peers reporting congruent habits), 2) both ego and alter non-drinkers, 3) both ego and alter reported any level of drinking but not weekly binge drinking, and 4) both ego and alter at least weekly binge drinkers. It is important to note that the variables on ego-alter similarity in alcohol use measure the co-occurrence of drinking habits, but it does not necessarily mean that egos and alters drink alcohol in the presence of each other.

#### Independent variables

Likewise, the dependent variables were based on egos’ responses and constructed based on similarity at the dyadic level. While the dependent variables regarded similarity in drinking habits, the independent variables indicated ego-alter similarity in relation to sex, age, ethnic background, religious beliefs, educational level and risk-taking. ‘Sex composition’ of the dyad distinguished between dyads of the same sex (‘male-male’ or ‘female-female’) and of ‘opposite sex’ (male-female). ‘Similar age’ accounted for possible age differences between ego and alter, and denoted whether the age gap was smaller or larger than three years. Ethnic background was categorized as dyads with similar ethnic backgrounds i.e., both peers had at least one parent who had migrated from former Yugoslavia or Iran, or both peers had parents with only Swedish descent, or peers had disparate ethnic backgrounds. Partly overlapping with this measure was the measure for religious beliefs/faith, which distinguished between dyads where both peers belonged to ‘disparate confessions and dyads where both peers were Christians (Protestant/Catholic), Muslims, belonged to another religious group (e.g., Mormons, Jehovah’s Witnesses, Jews) or where both peers were ‘non-religious’. Similarity in educational level referred to both ego and alter having completed at least secondary school. Another measure of similarity in peer-ties referred to risk-taking. The composite measure of ego-alter similarity in risk-taking was derived from two variables referring to whether ego was prepared to take risks and how strongly ego perceived that each alter in the ego’s network was to take risks. The new variable was coded “1” if ego agreed/strongly agreed to the statement of being a risk taker, and if ego also regarded alters as being risk-seekers. “Conformity” is a composite score reflecting the degree of similarity between ego and alter. The independent variables (i.e., ‘sex composition’, ‘similar age’, ‘similar educational level’, same ‘religious belief’, similar ‘risk taking’, and ‘discussing important matters with each other’) were used to create the index and each similarity counted as 1 point, resulting in a score with a range from 0 to 6.

Other types of social network characteristics comprised of the structure and content of the studied networks. ‘Type of relationship’ measured whether ego and alter were friends, relatives, or romantic partners, and ‘relationship duration’ referred to how many years ego and alter had known each other. ‘Network closure’, furthermore, referred to both the degree of closeness and the networks’ closure. Closed networks (coded as “1”) referred to all alters listed by ego knowing each other and networks which were more open (at least one missing link between alters) (coded as “0”). ‘Discussing important matters’ (coded as “1”) was used as an indicator of a strong relationship [10,50,51]. In addition, egos were asked about what types of topics that were commonly discussed among peers; ‘religion’ and ‘sex’ were two subjects reported to be more frequently discussed and are therefore included in the analysis as binary responses (1/0) and proxies for interest in faith, risk-taking and level of closeness in the dyad.

#### Statistical analysis

A hierarchical data structure with alters clustered by egos was applied. All components of the analysis refer to the dyad-level, which means that ego-alter ties (dyads) were treated as the smallest unit of analyses. Depending on the number of named alters, each respondent (ego) in the survey data contributed with up to five observations (7987 observations in total).

Multi-level multinomial logistic regression models with random effects (at ego-level) and 95% confidence intervals were used to estimate the relative risk ratios of dyadic alcohol habits by ego-alter (dis)similarity with regards to sex, age, ethnic background, religion, educational level and risk-taking, as well as other social network characteristics i.e., type of relationship, duration, network closure and closeness. A similar method with intra-class correlations to that used by colleagues [52] was used. The used two-level design with egos at the second level and alters at the first level accounts for the non-independence of alters clustered by egos.

To study interactions between demographic variables and network characteristics in relation to co-evolvement in alcohol habits, a predictive margins approach was used. Interactions between ethnic background and conformity, duration or density are illustrated in figures. Interactions between sex and ethnic origin were also analysed but were found to be non-significant and are therefore not presented further in this article.

## Results

To illustrate how network characteristics i.e., similarity in peers’ individual characteristics etc., relate to similarities in drinking habits in ego-alter dyads, both abstaining from alcohol, prevalence of drinking and frequency of binge-drinking was considered.

### Characteristics of peer dyads: Similarity, duration and closure, and type of relationship

The demographic characteristics of the sample illustrated in Table 1 show how peers tend to engage with peers similar to themselves. Male-male dyads and female-female dyads were more common than mixed dyads consisting of males and females. More than 85% of all ego-alter pairs were of similar age i.e., peers maximum three years younger/older. 23.4% of all ego-alter pairs did not have a similar ethnic background (both ego and alter parents from Sweden, former Yugoslavia or Iran). Most dyads had only secondary education, but 41.3% of the ego-alter pairs had also started tertiary education. Peer similarity was to some extent observed for the second measure of drinking cultures; 34.8% reported disparate religious beliefs to those of their peers. Similarity in risk- behaviours (23.7%) was also less pronounced than for the demographic aspects. In most dyads did both peers use alcohol (77.5%) and 9.2% of these ego-alter pairs binge drank at least once a week. Only 6.4% reported that neither them nor their peer were drinkers.

Most egos (83.3%) had listed friends (not relatives or romantic partners) as their closest peers. These relationships had, on average, prevailed for more than 8 years. The closure in these networks were also strong, given that in 74.2% of the dyads, all alters of the ego were friends with each other. Analyses of the types of subjects’ egos discussed with their peers (proxies for closeness in peer relationships) illustrated that 87.6% talked about important matters (general question). More specifically, 74.3% reported talking about sex life, and 52.6% talked about religion/faith.

### Alcohol habits in relation to network characteristics

A strong relationship could be observed between peers in dyads being non-drinkers and similarity in terms of them both being Muslims (Table 2). In concordance with this, if peers reported discussing religion with each other, both were more likely to be non-consumers of alcohol, whereas peers discussing sex with each other were less likely to be non-drinkers.

**Table 2:** Dyadic alcohol habits and social network characteristics: A two-level multinomial logistic regression model with random effects at ego-level (relative risk ratios with 95% confidence intervals).

Dyadic ego-alter variables	(1) Ego and alter never drink alcohol (concordance) vs (0) disconcordance in alcohol use		(2) Ego and alter drink alcohol (concordance) vs (0) disconcordance in alcohol use		(3) Ego and alter are weekly drunk (concordance) vs (0) disconcordance in alcohol use	
	RR	95% CI	RR	95% CI	RR	95% CI
Sex composition (Ref.: male-female)						
Male-male	0.99	(0.64–1.52)	1.34	(0.99–1.80)	1.74**	(1.20–2.52)
Female-female	1.26	(0.84–1.89)	0.74*	(0.55–0.98)	0.56**	(0.38–0.81)
Similar age	1.03	(0.74–1.44)	1.51**	(1.18–1.94)	1.79**	(1.25–2.56)
Ethnic background (Ref.: dissimilar ethnic backgrounds)						
Sweden	1.03	(0.71–1.50)	2.58**	(1.94–3.42)	4.09**	(2.88–5.82)
Iran	0.78	(0.51–1.19)	1.17	(0.84–1.61)	1.69*	(1.12–2.56)
Former Yugoslavia	1.06	(0.72–1.56)	1.42*	(1.04–1.93)	1.66*	(1.07–2.55)
Religious beliefs (Ref.: E&A disparate beliefs)						
E&A neither religious	1.13	(0.81–1.58)	2.26**	(1.79–2.86)	2.87**	(2.16–3.82)
E&A both Christian (Protestant/Catholic)	1.49	(0.91–2.45)	1.45	(1.00–2.10)	1.38	(0.86–2.20)
E&A both Muslim	4.86**	(3.18–7.43)	0.69	(0.47–1.02)	0.07**	(0.02–0.31)
E&A both other type of belief	1.27	(0.46–3.52)	1.33	(0.63–2.81)	0.46	NA †
E&A both risk-takers	0.89	(0.64–1.23)	1.26*	(1.00–1.58)	2.10**	(1.60–2.75)
E&A both started tertiary education	1.09	(0.79–1.50)	1.90**	(1.47–2.45)	2.27**	(1.68–3.05)
Type of relationship (Ref.: relatives)						
Romantic partners	1.80	(0.86–3.77)	1.16	(0.67–2.00)	0.23**	(0.10–0.54)
Friends (not relatives or romantic partners)	0.86	(0.53–1.38)	1.37	(0.95–1.97)	1.14	(0.66–1.95)
Relationship duration in years	1.01	(0.98–1.03)	1.00	(0.98–1.02)	0.95**	(0.93–0.97)
E&A in closed networks (Ref.: open networks)	1.02	(0.72–1.44)	1.91**	(1.43–2.55)	2.16**	(1.54–3.02)
Discusses important matters with each other	0.87	(0.59–1.26)	0.90	(0.69–1.16)	0.60**	(0.42–0.87)
Talk about religion	1.39*	(1.05–1.83)	0.90	(0.74–1.10)	0.89	(0.69–1.13)
Talk about sex	0.65**	(0.49–0.87)	2.23**	(1.79–2.78)	4.13**	(3.05–5.57)
*Variance (at ego-level)*			4.15 (Standard error: 0.39)			
Observations (ego-alter ties)			7,987			
Individuals (egos)			2,071			

* p < 0.05;

** p < 0.01.

^†^ Cannot be calculated due to the low number of cases.

Among drinkers (lower level), female peers were less prone to drink alcohol than those in mixed-sex (dissimilar) dyads. Non-religious peer dyads, in contrast, were more likely to be drinkers. Also, peers of Swedish descent and ego-alter pairs with roots in former Yugoslavia, were more likely to be drinkers than peers of dissimilar ethnic origin. Among peers who both had started tertiary education, it was also more common that they both were drinkers. Similarity in age also mattered, since ego-alter pairs of similar age were more likely to both be drinkers. Similarity in risk-taking behaviour among both peers also increased the risk of both peers being drinkers. Additionally, being members of a closed peer group was observed to increase the risk of being a drinker. Talking about sex among peers did also covariate with being a drinker.

In line with the results for prevalence of drinking, female and Muslim dyads were less likely to consume larger volumes of alcohol i.e., weekly binge drinking (subjective feeling of being intoxicated) were less common among these peers. Weekly binge drinking was found to be prevalent significantly more often in male and non-religious dyads in comparison to the mixed reference groups. Peers who had a similar ethnic background were additionally more likely to binge drink weekly than peers with dissimilar backgrounds. Similarity in age and educational level were two other demographics which also increased the risk of weekly binge drinking. For this riskier drinking behaviour, similarity in risk-taking behaviour among both peers were also observed to increase the risk. Binge drinking was additionally more common among peers who belonged to closed networks and among those who were close enough to talk about their sex life. Peers discussing important subjects more in general were, however, less likely to binge drink weekly. Type of relationship was also shown to be important, since dyads of romantic partners were significantly less likely to binge drink than dyads of relatives. Note that this was not the case for drinking in general.

**Fig 2:**
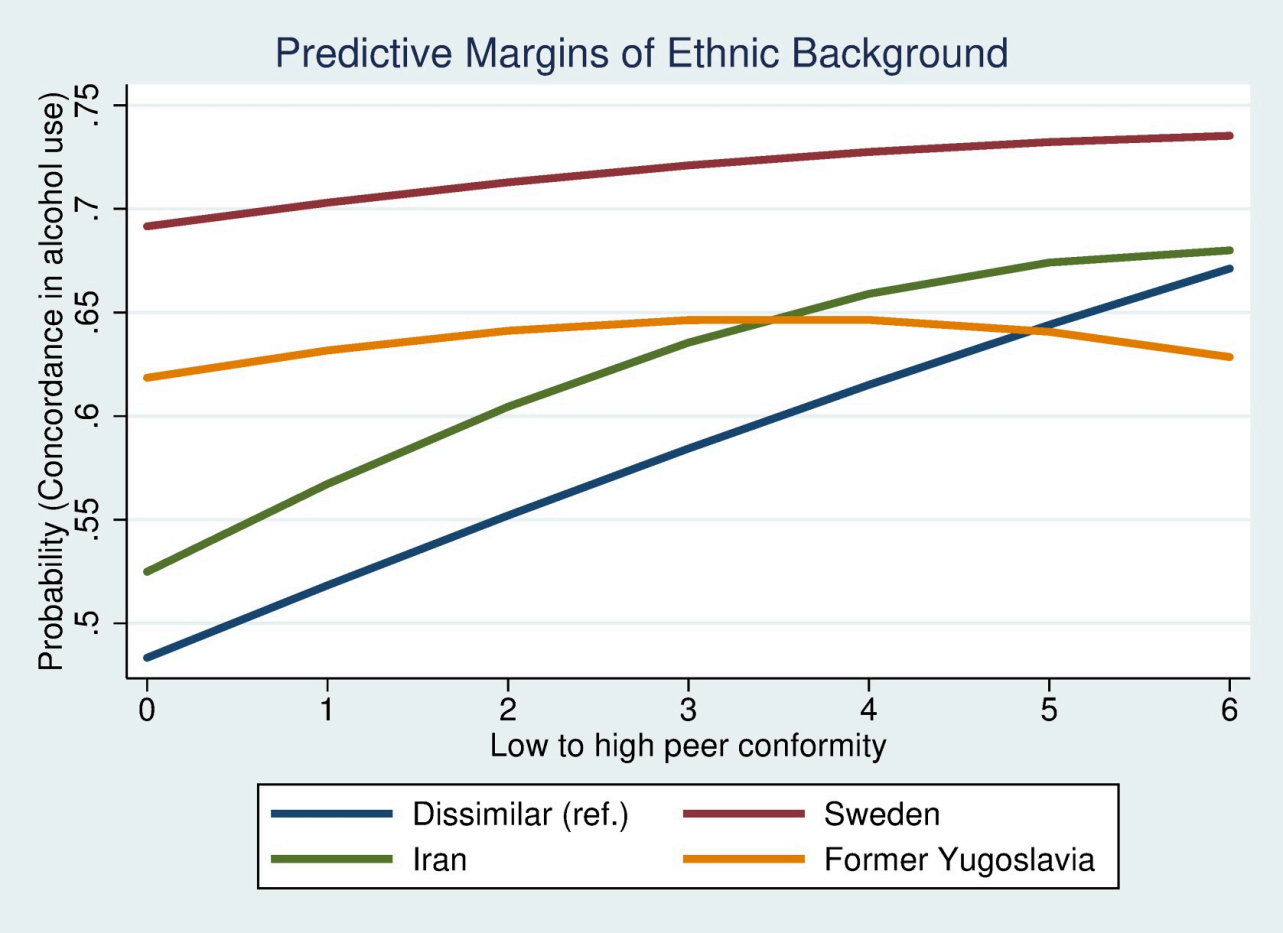
Concordance in alcohol habits by conformity in peer group.

**Fig 3:**
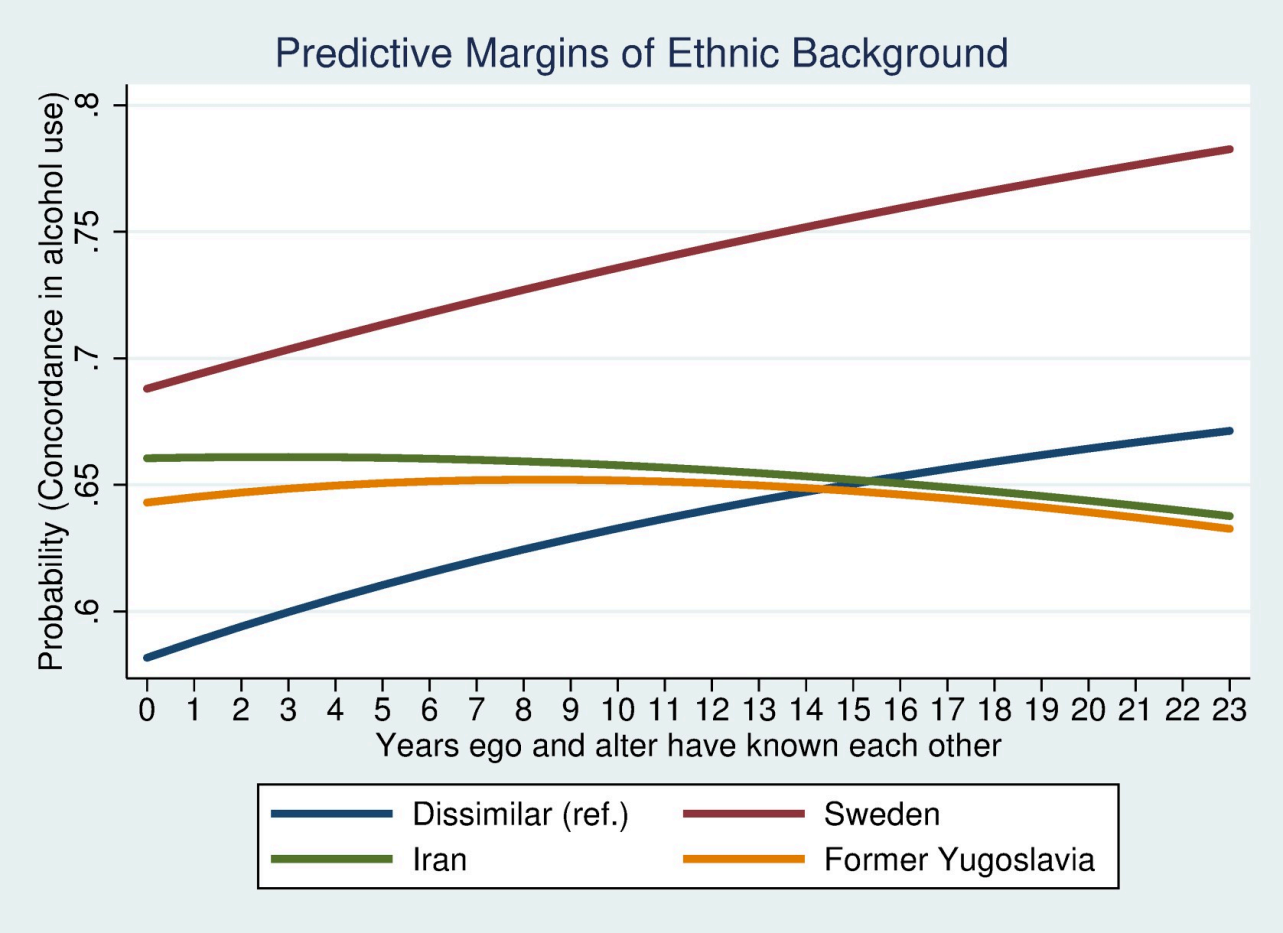
Concordance in alcohol habits by friendship duration.

**Fig 4:**
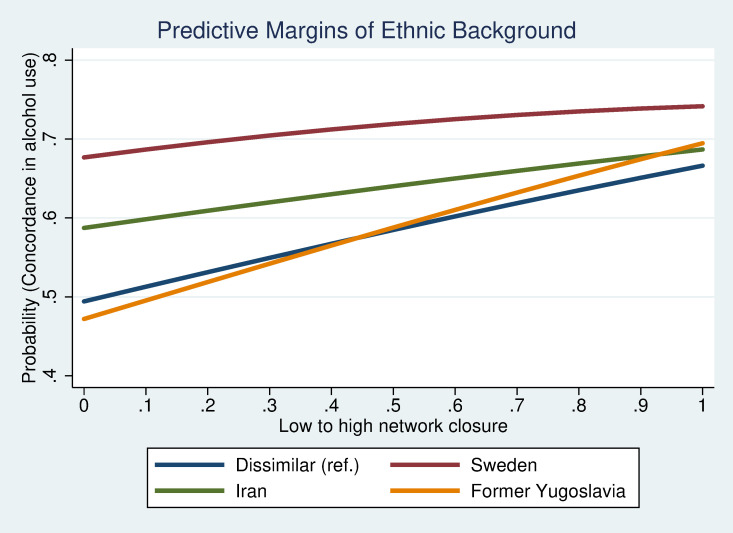
Concordance in alcohol habits by network closure.

The predictive margins plots illustrate that the concordance in drinking habits in relation to conformity in peer groups, duration of friendship and network closure differ between groups with different ethnic background. Fig 2 shows that those with parents from Sweden or former Yugoslavia, who are also likely to be more similar in their alcohol habits, is little affected by a higher conformity in the peer group. Peers with origin in Iran or a dissimilar background, on the other hand, show an increase in similarity in alcohol habits with increasing conformity between peers. Fig 3 illustrates how peers with dissimilar background or where both have a Swedish background, despite different initial level of similarity in habits, are more similar in their drinking habits the longer they have known each other. Alcohol habits among peers with a similar Iranian background seem to evolve in the opposite direction, although the change is not as drastic as for the two other peer groups. Peers with origin in former Yugoslavia do not seem to become more similar in their alcohol habits with time. Fig 4 shows that peers have more similar alcohol habits the more closed the network is. The trend for similarity in alcohol habits and network closure is similar regardless of ethnic background but seems to be particularly strong for peers with origin in former Yugoslavia. The results illustrate that estimates between dis-concordant and non-drinking do not differ that much. Concordant drinking, in contrast, seems to be driven by cultural/norm-based aspects and structural network characteristics. These explanations have an even stronger impact when it comes to binge drinking.

## Discussion

This study analysed how peer similarity in alcohol habits i.e., sobriety, prevalence of alcohol use and frequency of binge-drinking, relates to peer similarity in terms of gender, age, educational level, ethnic origin, religious beliefs and risk-taking. Peer similarity has been observed to be important for other health risk behaviours [18,19] and it was assumed that similarity (homogeneity) among peers would increase the likelihood of peers having similar norms and habits regarding alcohol as well. For the purpose of this study, a network structure in a Swedish sample of young adults and their peers was analysed at the dyadic level. People’s behaviours are usually influenced by the people they spend time with, and the closest peers were expected to have stronger impact over peer drinking than other people [10].

Two main incentives were assumed to drive peer clustering processes and the development of common drinking habits: norm-based aspects and contact opportunities. That peers similar to each other cluster partly happens early in life, when families with similar ethnic background, religious beliefs and socio-economic status tend to share more social arenas than families with larger differences [53–55]. Many activities during the school years are additionally structured around age and gender. Peers also select new friends based on similarity of demographic nature, personality traits e.g., risk-taking, norms and behaviours [10,11,14,56–59]. When peers consider themselves as “the same”, it can be expected that the step is shorter to also approach each other regarding other issues e.g., norm guided behaviours as studied in this article.

National drinking cultures have had a central role in research aiming to explain differences in alcohol habits between countries. Young people are known to be influenced by their parents drinking [31,60], and an assumption was made that the drinking culture and traditions from the parental country of origin, at least to some extent, would influence their children’s alcohol habits. This potential explanation has obtained more attention in Sweden since the early 2000’s as decreases in alcohol consumption have been observed, and it has been suggested that new migrant groups could explain parts of this change [61,62]. An additional indicator of drinking cultures and partly overlapping with the parental country of origin are the religious beliefs. Expectancies of less frequent alcohol use and lower consumption levels among people from Middle Eastern countries are funded on the religious restraints regarding substance use in Islam [26]. It was further assumed, based on the principle of homophily [10], that egos and peers who shared similar ethnic and or religious background would be more likely to select each other as friends. Ethnic similarity, based on parental country of origin, did seem to increase the risk of both being a drinker and binge drink weekly or more often (although non-significant for drinking in general among Iranians) than the group with mixed origin in the current study. It was unexpected that also ego-alter pairs with Iranian origin drank more alcohol than the mixed dyads. Given that people living in Middle Eastern countries are known to drink less [63], the current study hypothesized that also their offspring would drink less than the reference group with mixed origin. Pondering over the surprising results, it is possible that this second generation has been socialized into Swedish drinking habits [64]. It is also a possibility that Iranians are more secular (given their generally higher educational level) than most other populations in the Middle East, which could explain the higher alcohol consumption. Another possible explanation of this result is that other conditions in Sweden e.g., alienation, stigma, or lower socio-economic status, have contributed to higher alcohol use to cope. Still, the mixed group included people from a large variety of countries where some might not be too different from each other with regards to alcohol culture and others might be hugely different.

Peer dyads where both were Muslims were more often observed to abstain from alcohol than dyads with disparate religious beliefs. The Muslim dyads who did drink were much less likely to binge drink than the mixed group. Further in line with expectations was that peer dyads where both considered themselves to be non-religious were more likely to be drinkers and to binge drink than the mixed group. Faith in either part was on the contrary associated with a lower likelihood of peers in these dyads to be drinkers. While not all results were significant, peer clustering related to cultural norms in network dyads, and more specifically those related to drinking culture, were observed to be related to differences in alcohol habits.

As mentioned earlier, differences in drinking culture are not limited to national borders but can be observed also within a country [33], by sub-cultures or by population groups. Thus, we know that men and young people drink more than others [34,36]. In relation to demographic similarity, the expectation funded in empirical results on individual level was that dyads sharing these characteristics would also be more prone to drink and drinking larger amounts (binge drink). While the difference in prevalence of alcohol use was not observed to be large enough in male dyads compared to the mixed group (non-significant), male dyads were observed to get intoxicated more often. Female peer dyads were also less likely to be drinkers and to be frequent binge drinkers (at least weekly) than the mixed group.

Drinking was also hypothesized to occur in peer groups of similar (young) age. To assume a causal relationship to similarity and age would, however, be problematic since egos were 23 years old and peers of similar age here means that peers were between 20 and 26 years old. This result could instead be interpreted in the light of drinking being more common among younger people. Young people tend to spend time with other young people, due to similar lifestyles. This is increasing the contact opportunities with others part of this population group who is known to drink more alcohol than older people. This is likely to have impact on the common norms in the group and for peers to self-select to other groups with similar characteristics and behaviours. Given that they are likely to share these characteristics, drinking is more likely to occur, and binge-drinking is furthermore more likely to take place in party settings, assumed to be more frequent in this age group. As people grow older, lifestyles can change, resulting in changes of opportunities to drink as well as changes in norms, with the consequence that people tend to drink less alcohol. A dyad of two older people of similar age would therefore be expected to drink less alcohol than the group of young people in this study.

Education level was partly associated to age since many in this group of young people have not yet finished their studies. Although, the dyads where both peers had started some tertiary education were more likely to be drinkers as well as more often binge drink weekly compared to other dyads. Campus environments are known to encourage alcohol use [65], thus contributing with opportunities to drink.

Both ego and alter being identified as risk-takers furthermore increased the risk for dyads to binge drink at least weekly, which was also what we would expect from other studies on risk-taking and alcohol [66].

Based on the joint findings of similarity in peer dyads, similarity as an explanation of similar alcohol habits seems to be fairly strong. Dyads of peers being members of population groups known to more often be drinkers in individual-level studies were also found to be drinkers more often, and to drink larger amounts at single occasions (binge drink). This suggests that people who are similar in other aspects also tend to share similar norms and behaviours about whether to use alcohol at all or specifically to encourage binge drinking.

Other general social network characteristics i.e., relationship type, network closure, friendship duration and closeness in ties could have an impact on similarity and contact opportunities. With greater contact opportunities, peers are also more likely to share similar norms and behaviours regarding use of alcohol. The intensity in the relationship was for example expected to increase the number of contact opportunities, which was assumed to make peers more likely to share similar norms regarding drinking. In their early twenties, people tend to spend more time with other peers than with relatives and given that alcohol use is a social activity, alcohol use is likely to take place during some of these occasions and contributing to more drinking opportunities. Drinking among friends were not found to differ significantly from that among relatives but drinking among romantic partners did. Dyads of romantic partners were less likely to binge drink weekly than dyads of family members. Partners have been observed to have a central role in pressuring their partners to drink less [45]. This is additionally more common in so-called “dry” drinking cultures where per capita consumption is low due to less frequent drinking whilst binge drinking is commonly observed during weekends [67]. Sweden is one of the stereotypical countries with this type of drinking culture. For a person with higher consumption than their partner, the situational norms could involve pressure to drink less when spending time with their other half of the romantic dyad. Thus, it is likely that men in relationships more often were the ones who decreased their higher consumption when spending time with their romantic partners.

Close friendships also seemed to be important when explaining differences in drinking habits, since binge drinking were less common in peer dyads where important matters were usually discussed. Studies have shown that close friends influence drinking and have additionally suggested that ties are closer among peers sharing similar habits [68–70]. Considering two personal subjects commonly discussed in dyads, religion, and sex, it could be observed that peers in close friendship unions of less conservative nature i.e., discussing sex, were more prone to be drinkers as well as to binge drink weekly. This particular result should be interpreted with caution. Although one could expect certain discussion topics i.e., sex life, to be reflected upon more often among peers who have a closer relation, this subject is probably also more likely to come up in a drinking context when people are intoxicated. Peers discussing religion were more likely to be non-drinkers, which was partly consistent with the results of similar religious beliefs (for Muslims only). This further implies that peers with close ties are more likely to influence each other’s drinking norms but depending on the norm it can either increase or decrease alcohol use.

Additionally, if peers were part of a closed network, they were more likely to be drinkers and to binge drink at least weekly. Other health behaviours, positive or negative, have also been explained by stronger joint norms being upheld in closed networks, and what more that the norms in closed networks, once developed between peers, are harder to change [49]. Any policy intervention should therefore be carried out during an early stage of this process.

A limitation of this study is the focus on dyads and that the information about the extended network is limited. It is possible that ‘dyadic effects’ and ‘group effects’ work simultaneously to influence drinking in peers. Although, as stated in the introduction, the closest dyads are expected to have the strongest impact on behaviours. Still, in order to study the effects of the complete network, information about all peers and how they are connected would be needed. Another limitation with the analyses is that data was ego-based and thus only providing second-hand information from egos (secondary source) regarding peers’ drinking. Several studies have stated that there is a tendency that people overestimate peers’ drinking, although the accuracy of this conclusion has also been questioned [71]. Such misreporting of peers’ behaviours could induce that the link between ego and alter behaviour appears stronger than it is. The error is likely to be stronger if the ego-alter link is weak [18]. A preferred strategy to overcome misreporting is the use of a complete sociocentric network design that includes multiple reports from both egos and alters [72,73]. With the potential risk introduced in this study by egos’ not estimating their peers drinking habits correctly, it can still be argued that the perceived drinking and norms of peers is what affects the own drinking. This study did, furthermore, not investigate peer selection, or whether similarity in norms or alcohol habits comes first. The ego-based data limit the possibilities of ruling out whether peer effects are confounded by peer selection. Methods to identify peer effects would for example be the use of instrumental variables [74,75]. Longitudinal data would be needed for drawing causal conclusions, but crucial variables were only captured in the second wave of the data collection. It is, however, outside the scope of this article to answer the question of what matters most and this explorative study rather focused on whether similarity in peer dyads and other network characteristics could explain similarities in alcohol habits. A further limitation in this study is that the size of the sample is only large enough for three groups based on ethnic origin. Given that the study was conducted in Sweden, the mixed origin group was also more likely to include Swedes. No information is available for how long parents have lived in Sweden, but this is important for acculturation processes and to what extent parents have adapted to Swedish drinking culture. Another drawback is that data does not consider situational aspects. This study cannot claim for certain that drinking occurred when peers were spending time together or that discussions about certain subjects took place during drinking sessions e.g., parties, with their peers. For that reason, it is not possible to study the impact of competing explanations of behavioural copying or imitation. During drinking situations, it is furthermore possible that drinking is driven by a single heavy consumer influencing the peer or group of peers [2]. The complexity of situational drinking [3] is an area which should be explored further in future studies.

In conclusion, the results in this study suggest that network similarity i.e., homophily, and other network characteristics are important when explaining co-existence of both prevalence of alcohol use and drinking patterns i.e., frequency of binge drinking, in dyads of peer networks. Knowing that people are connected through social network ties has bearing on policy development since interventions are more successful if there is similarity in the intervention group and if they also target spouses or peers [5]. Policies targeting the whole population have been successful and should not be understated, but when targeting certain population groups, the advice to policy makers is to consider peer similarity as an additional aspect in planning and evaluating interventions on harmful alcohol use. One can e.g., expect that interventions targeting schools are less successful if the group of pupils is remarkably diverse regarding migration background and other sociodemographic aspects.
